# Assessing the diagnostic performance of the muscle biopsy features in the 2024 ENMC Inclusion Body Myositis Criteria

**DOI:** 10.1093/rheumatology/keag244

**Published:** 2026-05-11

**Authors:** Mustafa Ekici, James B Lilleker, Hector Chinoy

**Affiliations:** Department of Rheumatology, Salford Royal Hospital, Northern Care Alliance NHS Foundation Trust, Manchester Academic Health Science Centre, Salford, United Kingdom; Division of Musculoskeletal and Dermatological Sciences, Faculty of Biology, Medicine and Health, The University of Manchester, Manchester, United Kingdom; Department of Internal Medicine, Division of Rheumatology, Ankara Bilkent City Hospital, Ankara, Turkey; Manchester Centre for Clinical Neurosciences, Salford Royal Hospital, Northern Care Alliance NHS Foundation Trust, Salford, United Kingdom; Department of Rheumatology, Salford Royal Hospital, Northern Care Alliance NHS Foundation Trust, Manchester Academic Health Science Centre, Salford, United Kingdom; Division of Musculoskeletal and Dermatological Sciences, Faculty of Biology, Medicine and Health, The University of Manchester, Manchester, United Kingdom

Rheumatology key message

- The 2024 ENMC criteria showed high sensitivity, with nearly all cases classified by the 2013 criteria also meeting the updated criteria.

Dea r Editor, Inclusion body myositis (IBM) is the most common acquired myopathy with symptom onset above approximately age 45 years. The 2013 European Neuromuscular Center (ENMC) diagnostic criteria for IBM specify a clinicopathological approach based on demonstration of a typical weakness pattern, together with key muscle biopsy features. However, in clinical practice, early or atypical cases may be identified without sufficient features to fulfil these criteria and may thus receive suboptimal care or be offered inappropriate treatment [1]. Furthermore, requiring the presence of clinical and pathological features associated with significantly established disease may limit the potential effectiveness of any investigational therapeutics due to the presence of irreversible tissue damage.

The updated 2024 ENMC criteria seek to address these issues by proposing a simple two-step process: firstly, to define the type of clinical presentation, which now also accounts for atypical presentations (e.g. isolated dysphagia), and secondly, to identify supportive investigation results, with a sliding scale of requirements according to the nature of the clinical presentation (typical *vs* atypical). Endomysial inflammation remains mandatory; supportive investigations include muscle MRI/ultrasound, anti-cN1A testing, and mitochondrial pathology [2]. The new criteria also define patients dichotomously as meeting the diagnostic criteria or not, unlike the previous criteria, which had a hierarchical system.

We aimed to evaluate the performance of the 2024 ENMC diagnostic criteria for IBM, compared with the 2013 criteria, among patients with IBM followed up in our clinic. All included patients had an expert-defined diagnosis and an available muscle biopsy. We conducted a retrospective case note review at the Adult Neuromuscular Clinic, Salford Royal Hospital, a regional tertiary neuromuscular referral centre serving North West England. Our study included all eligible patients seen between 2004 and 2024. This work was conducted as a service evaluation project, and ethics approval was not required. As the data were collected retrospectively from hospital records, no separate patient consent was obtained.

Initially, a total of 88 patients were identified from clinic lists and local databases. Two incorrectly coded patients were excluded after initial casenote review. Four other cases were excluded: two who were initially thought to have IBM but where alternative diagnoses were identified during follow up (1 patient with myotonic dystrophy type 2, and one with a mitochondrial myopathy); and two due to non-diagnostic muscle biopsies (advanced fibrosis and fatty replacement, making sufficient analysis to apply the criteria impossible), where a clinical diagnosis of IBM had been made.

We then applied the ENMC 2013 and ENMC 2024 diagnostic criteria to each remaining case (*n* = 82) using published definitions and all available follow-up information contained within the casenotes. Non-classified cases, or those who met one of the diagnostic criteria but not the otherwere independently re-reviewed by a second experienced specialist (Fig. 1).

**Figure 1:**
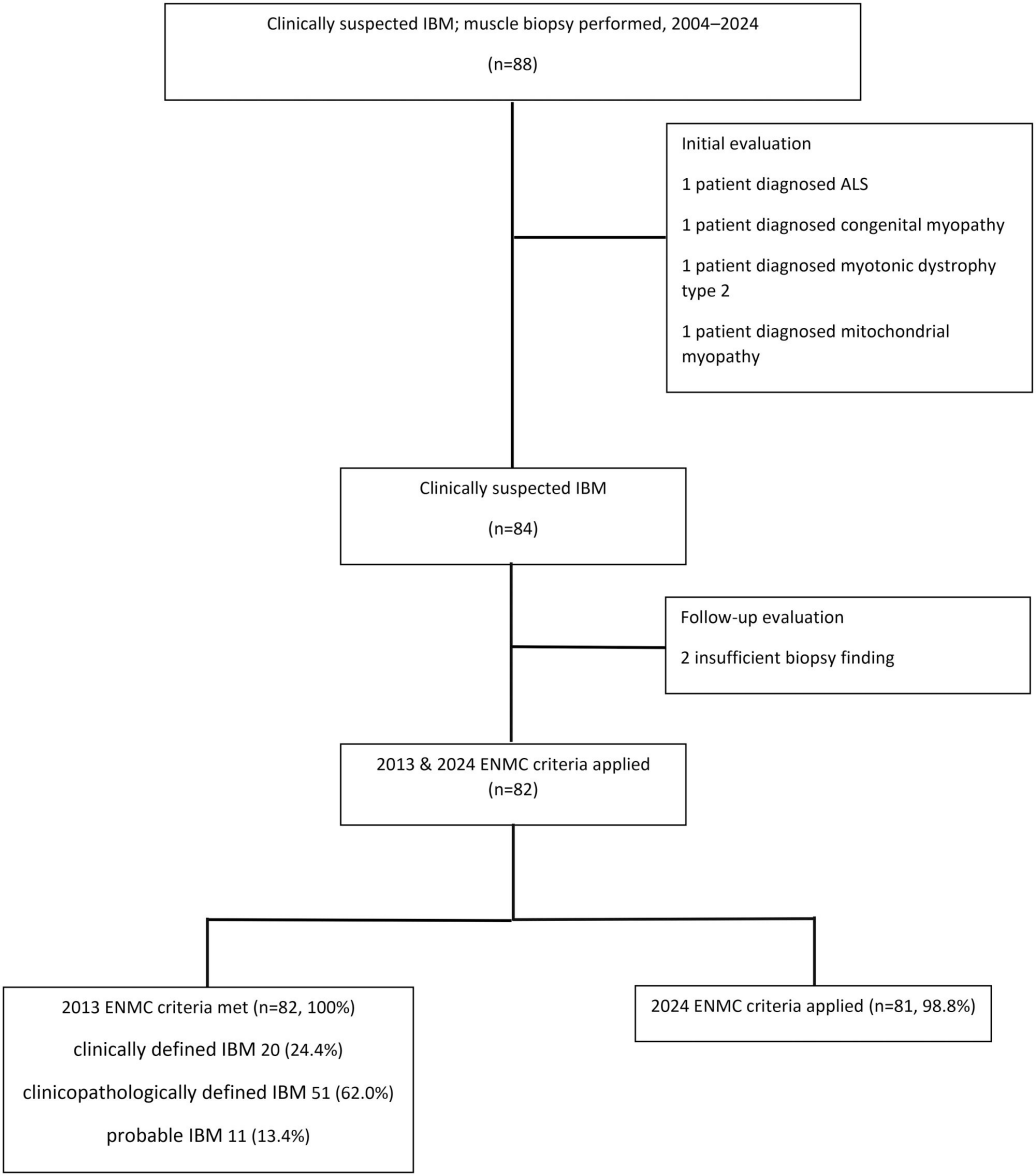
Patient selection and classification according to the 2013 and 2024 ENMC criteria

According to the 2013 criteria, 82/82 patients (100%) fulfilled at least the ‘probable’ criteria. Of these, 20 patients (24.4%) were classified as having ‘clinically defined’ IBM, 51 (62.0%) as ‘clinicopathologically defined’ IBM, and 11 (13.4%) as having ‘probable’ IBM. Using the 2024 criteria, 81/82 (98.8%) met the diagnostic threshold.

Of the 82 patients, 52 (63.4%) were male, with a mean age of 67.0 ± 7.6 years. Using the 2013 criteria as the reference standard, the 2024 criteria showed a sensitivity of 98.8% (81/82; 95% CI 93.4–99.8). In the case that did not fulfil the 2024 ENMC criteria, the primary reason was the absence of endomysial inflammation on biopsy despite typical clinical features and quadriceps-predominant MRI changes. The 2013 ‘probable’ criteria were met for this patient due to the presence of some sarcolemmal HLA-1 expression.

Of the patients tested for anti-cN1A autoantibodies, 7/29 (24.1%) were antibody positive. In none of these cases was anti-cN1A positivity required as the sole determinant to satisfy the classification criteria.

In our biopsy-based cohort, the 2024 ENMC IBM criteria showed very high sensitivity. This is higher than that of prior studies, which have shown uniformly high specificity but widely varying sensitivity (11–84%), potentially reflecting our case mix as a research-active referral centre [3]. The ‘false-negative’ case, according to the 2024 criteria, had a typical clinical phenotype but without endomysial inflammation on muscle biopsy. Real-world factors, such as biopsy timing and sampling variability related to muscle choice, may explain this finding. In this patient, a vastus lateralis biopsy was obtained 2.6 years after clinical diagnosis, and the patient had not received immunosuppressive therapy before the biopsy. Of note, this case still had supportive investigation findings, in addition to the typical initial clinical features: quadriceps-predominant MRI changes, MHC class I upregulation, sarcoplasmic p62 aggregates, rimmed vacuoles, and mitochondrial abnormalities [1, 2, 4–6].

As anticipated during the formulation of the 2024 ENMC diagnostic criteria, removal of the ‘probable’ IBM category and the mandatory requirement to demonstrate the presence of an inflammatory infiltrate make the new criteria somewhat more stringent. However, the reduction in sensitivity we observed in this study is very small and is likely to be mitigated by the ability of the new criteria to use muscle imaging and anti-cN1A antibody status to support a diagnosis of IBM, although we were not able to demonstrate this in our study. Further prospective study will be required to understand the full potential of the new criteria to increase sensitivity.

Our study provides reassurance that the vast majority of patients diagnosed using the 2013 criteria also meet the 2024 criteria, and that the pool of patients potentially able to take part in IBM clinical trials remains undiminished. Given the skewed nature of our cohort and the long-term application of the 2013 criteria, it is likely that the full potential benefits of the new criteria have not been demonstrated.

## Data Availability

The data underlying this article will be shared on reasonable request to the corresponding author.
